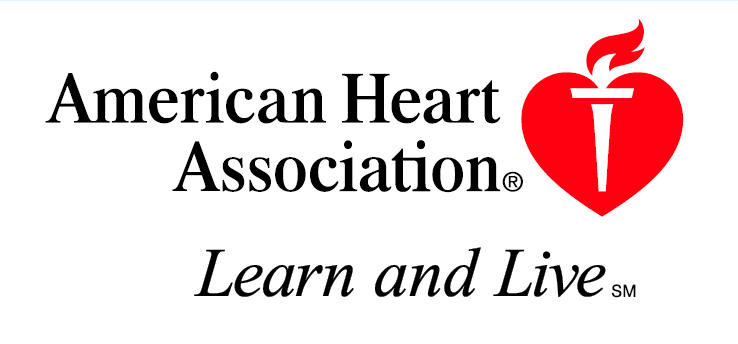# EHPnet: American Heart Association

**Published:** 2004-11

**Authors:** Erin E. Dooley

Many diseases fall under the umbrella of “cardiovascular disease,” including high blood pressure, cardiac arrhythmia, and congenital heart disease. These conditions affect young and old alike, as well as every ethnic group. In the United States, heart disease is the leading cause of death for both men and women. In 2004 alone, it is expected to cost the U.S. economy over $238 billion in health care services, medications, and lost working hours. Since 1924 the American Heart Association (AHA) has been working to raise funds for heart disease research and to generate awareness among the general public about the seriousness of these diseases. The AHA website located at **http://www.heart.org/** disseminates information about heart disease and the association’s wide range of public information and continuing education programs.

A menu bar down the left-hand column of the homepage includes a pull-down listing of 10 diseases and conditions, which can then be selected for in-depth information. For example, the page on high blood pressure provides facts on the disease, ways to keep it in check, how to sign up for the AHA’s monthly e-mail list service, and more. Visitors can also find information on risk factors, how a condition can affect health, and information geared toward medical professionals.

Another option on the menu bar leads to information on heart disease specifically in children. Along with a basic overview of childhood heart disease, this section includes in-depth pages on topics including DiGeorge syndrome, Kawasaki disease, and exercise for children. Also accessible from the children’s section is HeartPower!, a free, downloadable curriculum devised to help teachers in grades pre-kindergarten through 8 teach their students about healthier lifestyles.

The Healthy Lifestyle link from the homepage leads to information on topics ranging from diet and nutrition to heart disease in women. Various pages provide tips on creating healthy eating habits, developing and maintaining food plans, reducing cholesterol, and keeping fit. The Health Tools portion of this section has a cardiovascular disease risk assessment tool; a family health history tree; and online logs for tracking blood glucose, blood pressure, cholesterol, and exercise.

The Publications section provides links to lists of AHA-produced consumer and patient education materials, many of which can be ordered for free. Visitors will also find a listing of AHA cookbooks (with sample recipes posted online) and other books on heart health. The AHA publishes five journals that also can be accessed online, as can a variety of other scientific publications, including Scientific Statements from the AHA, performance measures, and clinical data standards. The online Heart and Stroke Encyclopedia is available from the homepage as its own section.

Visitors to the homepage will find information on current events, and can search by zip code for nearby events and information on local AHA chapters. Many of the site’s pages are available in Spanish, and can be accessed through the En Español link on the homepage.

## Figures and Tables

**Figure:**